# Missing flux from the ocean: isotope revealed an important NO_x_ flux mediated by microbial processes

**DOI:** 10.1093/nsr/nwac142

**Published:** 2022-07-27

**Authors:** Keisuke Koba

**Affiliations:** Center for Ecological Research, Kyoto University, Japan

NO_x_ in the atmosphere can affect human health and economic development as well as atmospheric chemistry and climate. Thus, it is crucial to deeply understand the dynamics of NO_x_ including its sources and sinks. However, as in the cases of other nitrogen compounds such as nitrous oxide, it is quite difficult to trace the fate of NO_x_, then close its budget.

Song *et al*. [1] nicely tackled this issue with the help of the ^15^N signature of NO_x_. They summarized the *δ*^15^N data of NO_3_^−^ in atmospheric particulates observed in the ocean sites, which are quite low. Even after the correction of the potential changes in *δ*^15^N, the low *δ*^15^N signatures of ocean NO_x_ (−12.5‰ on average) cannot be explained by *δ*^15^N of NO_x_ from the oil combustion of marine traffic transportation (+4.7‰ on average), which has been considered as a single NO_x_ emission source in marine environments. The simple isotope mass-balance model required them to include one more NO_x_ emission source with low *δ*^15^N: microbially derived NO_x_.

The contribution of microbial NO_x_ emission was estimated as 8.8 ± 1.5 Tg-N/yr [1]. Compared to the total ocean NO_x_ emission (15.2 ± 1.5 Tg-N/yr), this flux estimated by Song *et al*. [1] is shockingly high. However, this estimation looks reasonable when compared with other important nitrogen fluxes in the ocean such as denitrification (100–450 Tg-N/yr [2]), nitrous oxide emission (3.4 Tg-N/yr [3]) and nitrification (100–200 Tg-N/yr [4]). Many microbial processes such as nitrification and denitrification are known to produce NO, the precursor to atmospheric NO_x_. Song *et al*. [1] appealed the urgent necessity for more research on the microbial NO_x_. It should be noted that although the *δ*^15^N signature of NO_x_ is quite powerful in elucidating the missing NO_x_ fluxes as found in Song *et al*. [1], the estimation, inevitably, heavily depends on the endmembers *δ*^15^N data of NO_x_ and isotopic changes during the formation of particle NO_3_^−^, both of which are quite difficult to obtain and to constrain. More intensive research on the production/consumption of NO_x_ as well as isotopic fractionations during these processes can make great contributions to constraining the microbially derived NO_x_ fluxes in the ocean and land that are now beautifully unlocked by Song *et al*. [1].
